# DIET AND NUTRITION: Next Course in Organic Debate

**Published:** 2009-10

**Authors:** David C. Holzman

**Affiliations:** **David C. Holzman** writes from Lexington and Wellfleet, Massachusetts, on science, medicine, energy, economics, and cars. He has written for *EHP* since 1996

With the Northern Hemisphere’s peaking summer produce crop came a new iteration of the question of whether organic food is worth the extra expense. According to a review commissioned by the U.K. Food Standards Agency and published 29 July 2009 ahead of print in the September 2009 *American Journal of Clinical Nutrition*, organically grown food is no more nutritious than conventionally grown food. But organic proponents question the findings and also note that the health benefits of organic agriculture can go beyond nutrition.

The review authors identified more than 52,000 studies dating back to 1958 that compared organic and conventional foods. Of these, 55 studies were deemed of sufficient quality. None of the studies predate 1990—an important point, says first author Alan D. Dangour, a senior lecturer at the London School of Hygiene & Tropical Medicine, because one chief criticism of the review has been the fact that agriculture has changed markedly over the past 60 years.

Of 11 parameters examined, organic crops had significantly higher phosphorus content and titratable acidity (which is not a nutrient but a food processing metric) whereas conventional crops had higher nitrogen content. Differences in levels of vitamin C, phenolic compounds, magnesium, calcium, potassium, zinc, total soluble solids (mostly sugar), and copper were insignificant.

The results contradict a review published as the report *New Evidence Confirms the Nutritional Superiority of Plant-Based Organic Foods* in March 2008 by The Organic Center, a nonprofit food research outfit. This review found that total phenolics, vitamin E, vitamin C, quercetin, and total antioxidant capacity of organics exceeded that of conventionally grown produce—in the case of total antioxidant capacity, by 80%. Conventional products had higher levels of potassium, phosphorous, and total protein, all basic constituents of conventional fertilizers. Nutrition scientist Denis Lairon reported similar findings in a review published online 8 July 2009 ahead of print in *Agronomy for Sustainable Development*.

Charles Benbrook, The Organic Center’s chief scientist and coauthor of the *New Evidence* report, criticizes the U.K. review for not requiring the individual studies to have used the same cultivars on organic and conventional plots. Differences in plant varieties—for instance, between hybrid and heirloom varieties of tomatoes—can result in dramatic differences in nutrient content, he says. “We went with what was available,” responds Dangour. “In general I recall most studies compared the same cultivars.”

Another criticism: the study did not require organic fields to have been used as such for a minimum number of years. “We know from a large body of research that the biological benefits of organic farming mostly come from improvements in soil quality,” says Benbrook. Moreover, long‐term studies of organic and conventional tomatoes by food chemist Alyson Mitchell at the University of California, Davis, have demonstrated that soil organic matter takes at least five years to reach optimal levels. And national standards defining organic production practices were not established until 2002.

Mitchell cautions against drawing sweeping conclusions from comparison studies of organic versus conventional. She notes that organic practices vary immensely, with some modern industrial‐scale organic farms using methods more similar to those of conventional farms, such as growing just a single crop (monoculture).

But Benbrook says sustainably conducted organic farming offers benefits beyond nutrition, including improved health of pollinators and cleaner waterways resulting from minimal pesticide use, significant carbon sequestration as soil builds up through organic cultivation, and potential shrinkage of oceanic dead zones due to reduced nitrogen fertilizer pollution. Melissa Perry, an associate professor of occupational epidemiology at the Harvard School of Public Health, also says children on organic diets have shown significantly lower levels of pesticide metabolites in their urine than children on conventional diets. As to the dangers thereof, Perry says the risk assessments conducted so far by the Environmental Protection Agency are limited because they do not routinely account for cumulative and potentially synergistic effects of multiple pesticides.

